# *DoGMP1* from *Dendrobium officinale* contributes to mannose content of water-soluble polysaccharides and plays a role in salt stress response

**DOI:** 10.1038/srep41010

**Published:** 2017-02-08

**Authors:** Chunmei He, Zhenming Yu, Jaime A. Teixeira da Silva, Jianxia Zhang, Xuncheng Liu, Xiaojuan Wang, Xinhua Zhang, Songjun Zeng, Kunlin Wu, Jianwen Tan, Guohua Ma, Jianping Luo, Jun Duan

**Affiliations:** 1 Key Laboratory of South China Agricultural Plant Molecular Analysis and Gene Improvement, South China Botanical Garden, Chinese Academy of Sciences, Guangzhou 510650, China; 2 University of Chinese Academy of Sciences, Beijing 100049, China; 3 P. O. Box 7, Miki-cho post office, Ikenobe 3011-2, Miki-cho, Kagawa-ken, 761-0799, Japan; 4 School of Food Science and Engineering and Biotechnology, Hefei University of Technology, Hefei 230009, China

## Abstract

GDP-mannose pyrophosphorylase (GMP) catalyzed the formation of GDP-mannose, which serves as a donor for the biosynthesis of mannose-containing polysaccharides. In this study, three *GMP* genes from *Dendrobium officinale* (i.e., *DoGMP*s) were cloned and analyzed. The putative 1000 bp upstream regulatory region of these *DoGMP*s was isolated and *cis*-elements were identified, which indicates their possible role in responses to abiotic stresses. The DoGMP1 protein was shown to be localized in the cytoplasm. To further study the function of the *DoGMP1* gene, *35S*:*DoGMP1* transgenic *A. thaliana* plants with an enhanced expression level of *DoGMP1* were generated. Transgenic plants were indistinguishable from wild-type (WT) plants in tissue culture or in soil. However, the mannose content of the extracted water-soluble polysaccharides increased 67%, 96% and 92% in transgenic lines #1, #2 and #3, respectively more than WT levels. Germination percentage of seeds from transgenic lines was higher than WT seeds and the growth of seedlings from transgenic lines was better than WT seedlings under salinity stress (150 mM NaCl). Our results provide genetic evidence for the involvement of *GMP* genes in the biosynthesis of mannose-containing polysaccharides and the mediation of *GMP* genes in the response to salt stress during seed germination and seedling growth.

GDP-mannose pyrophosphorylase (GMP, E.C. 2.7.7.13), also known as mannose-1-phosphate guanyltransferase, catalyzes the conversion of mannose-1-phosphate to GDP-mannose. A number of nucleotide sugars such as GDP-L-galactose, GDP-L-fucose and GDP-D-rhamnose are synthesized using GDP-mannose as the precursor^1^,^2^. Moreover, GDP-mannose is an important intermediate product related to a wide range of metabolic pathways in plants, such as N-glycosylation^3^,^4^ and the synthesis of ascorbic acid (AsA) and polysaccharides^1^. Insertion of the *GMP* gene into *Saccharomyces cerevisiae* restored the viability of *alg1* N-glycosylation mutants^5^. GDP-mannose deficiency, which is caused by GMP deficiency, is responsible for N-glycosylation deficiency, and results in inhibited root growth in the presence of NH_4_^+^ 6,7.

In plants, the *GMP* gene is involved in AsA synthesis and has been shown to improve the tolerance of plants under abiotic stress. For example, in an ozone-sensitive *Arabidopsis thaliana* mutant (*sozi*/*vct1*/*cyt1*) that contained only 30% of the wild-type (WT) AsA concentration, the *VCT1* gene, which encodes a GMP^1^, was shown to be responsible for AsA deficiency^8^,^9^. *GMP* from rice (*Oryza sativa* L.) improved salinity stress tolerance in tobacco (*Nicotiana tabacum* L.)^10^. A tobacco *GMP* is involved in tolerance to temperature stress^11^,^12^.

Previous studies demonstrated that mannan synthase isolated from plant species such as pea (*Pisum sativum* L.), fenugreek (*Trigonella foenum*-*graecum* L.) and guar (*Cyamopsis tetragonoloba* L.), used GDP-mannose as a substrate to synthesize a mannan backbone *in vitro*^13^,^14^. The cellulose synthase-like A (*CSLA*) family, which belongs to the cellulose synthase (*CesA*) superfamily of glycosyltransferase family 2 (GT2), encodes proteins responsible for mannan polysaccharides by using GDP-mannose as the substrate^15^,^16^,^17^. Transformants of potato (*Solanum tuberosum* L.) with reduced GMP activity had 30–50% lower mannose content than WT plants^4^.

Although the vast majority of the characterized *GMP*s from *A. thaliana*, rice or other higher plant species have been analyzed, it has been proposed that the involvement of *GMP*s in AsA synthesis and stress tolerance is conserved. However, it is not possible to predict gene functions of possible *GMP*s based only on nucleotide or amino acid sequence similarities. For example, a probable GDP-mannose pyrophosphorylase (TAIR number: AT1G74910), predicted by amino acid sequence similarly, lacks GDP-mannose pyrophosphorylase activity^18^. In this study, we report on the cloning and characterization of three GMPs from *D. officinale*. We generated *35S*:*DoGMP1 A. thaliana* transgenic lines, studied the relationship between *DoGMP1* and mannose content of water-soluble polysaccharides, and assessed the tolerance of these lines to salinity stress. This work can provide insight into understanding the molecular mechanisms of polysaccharide biosynthesis in *D. officinale*. Furthermore, this work also has implications for the development of abiotic stress-tolerant crops to overcome environmental stress limitations and improve production efficiency in the face of a burgeoning world population.

## Materials and Methods

### Plant materials and growth conditions

Potted *D. officinale* plants used to clone genes were grown and maintained in a greenhouse (Guangzhou, Guangdong, China) under natural conditions. The stems of *D. officinale* (about one year old) were harvested, frozen rapidly in liquid nitrogen and kept at −80 °C until RNA extraction. *A. thaliana* (ecotype Columbia) was used as the WT in this study. WT and *DoGMP1* overexpression lines were cultured in a growth chamber in a 16-h photoperiod (100 μmol m^−2 ^s^−1^) at 22 °C. Plants were grown in pots (8 × 10 cm, diameter × height) filled with soil (topsoil and vermiculite; 1:2) and watered periodically with Hyponex fertilizer (N:P:K = 6-10-5, diluted 1,000-fold; Hydroponic Chemicals Co., Ohio, USA).

### Cloning GDP-pyrophosphorylase genes from *D. officinale*

According to the annotation of unigenes of an in-house transcriptome reference database of sequences^19^, GDP-mannose pyrophosphorylase unigenes were identified and used to design primers. The total RNA of *D. officinale* was isolated by using Column Plant RNAout2.0 (Tiandz, Inc., Beijing, China) according to the manufacturer’s protocol. Two μg of total RNA were reverse transcribed for the first-strand cDNA, which served as the template to generate 5′ and 3′ cDNA ends by using M-MLV reverse transcriptase (Promega, Madison, Wisconsin, USA). The SMARTer^TM^ RACE cDNA Amplification Kit (Clontech Laboratories Inc., Mountain View, USA) was used to generate both 5′ and 3′ cDNA ends according to the manufacturer’s protocol. PCR products were purified by a Gel Extraction Kit (Dongsheng Biotech, Guangzhou, China), cloned into the pMD18-T vector (Takara Bio Inc., Dalian, China) and sequenced at the Beijing Genomics Institute (Shenzhen, Guangdong, China). Primer pairs for each gene designed to amplify 3′ and 5′ cDNA regions are listed in Supplementary Table 1.

### Isolation and analysis the putative promoters of *DoGMP*s

To understand the regulatory mechanism of *DoGMP*s, the Genome Walking Kit (Takara Bio Inc.) was used to clone the putative promoters of *DoGMP*s according to the user’s manual. Primers specific for each gene were designed by Primer Premier 5.0 (PREMIER Biosoft Palo Alto CA USA) and listed in Supplementary Table 2. The putative promoters were used to analyze the *cis-*regulatory elements by an on-line prediction soft (http://bioinformatics.psb.ugent.be/webtools/plantcare/html/).

### *DoGMP1*-*YFP* plasmid construction and localization analysis

The full-length coding sequences of the *DoGMP1* gene (excluding the termination codon) were amplified with a pair of primers (DoGMP1YFPF/DoGMP1YFPR, listed in Supplementary Table 3) introduced as adaptor sequences at the 5′ and 3′ ends according to the pSAT6-EYFP-N1 vector^20^ sequences. The principles of adaptor sequence design followed In-Fusion^®^ HD Cloning Kit (Clontech Laboratories, Inc.) instructions. The amplified product was inserted downstream of the 35S *Cauliflower mosaic virus* (CaMV) promoter in the unique *Nco*I site of the pSAT6-EYFP-N1 vector^20^ by using the In-Fusion^®^ HD Cloning Kit according to the manufacturer’s instructions. The *DoGMP1*-*YFP* recombinant plasmid was verified by DNA sequencing at the Beijing Genomics Institute. Transient transformation was performed with 10 μg of plasmid DNA transferred into mesophyll protoplasts from a 4–5 weeks-old *A. thaliana* plant by a polyethylene glycol (PEG)-mediated transfection system described by Yoo *et al*.^21^. Protoplasts were incubated for 20 h under standard light/dark conditions then yellow fluorescent protein (YFP) was localized via fluorescence microscopy. YFP fluorescence was visualized by a Zeiss LSM 510 confocal microscope (Zeiss, Jena, Germany).

### Construction of *DoGMP1* overexpression vector and *Arabidopsis thaliana* transformation

The full-length coding sequences of the *DoGMP1* gene (excluding the termination codon) were amplified and cloned into the pCAMBIA1302 vector at the *Nco*I site, driven by the 35S CaMV promoter. After verification by full sequencing at the Beijing Genomics Institute, the recombinant vector was transformed into *Agrobacterium tumefaciens* EHA105 by using the freeze-thaw method^22^ then used for *A. thaliana* transformation. Transgenic plants were generated by a floral dip transformation method^23^. The primer pairs designed for construction of *35S*:*DoGMP1* vector are listed in Supplementary Table 3.

### Western blot assay

Seven-day-old *A. thaliana* seedlings (0.5 g), grown on half-strength Murashige and Skoog medium^24^ containing 2% sucrose and 0.8% agar (pH 5.7) (basal medium, BM), were harvested and immediately ground in liquid nitrogen with a mortar and pestle. Cells were lysed in 700 μL extraction buffer (50 mM Tris-HCl at pH 7.4, 150 mM NaCl, 2 mM MgCl_2_, 1 mM dithiothreitol (DTT), 20% glycerol, 1% Nonidet P-40) containing a protease inhibitor cocktail (Cat. No. 04693132001, Roche, Basel, Switzerland), then centrifuged at 4 °C and 14,000 g for 20 min. The supernatants containing total proteins were fractionated by SDS-PAGE and analyzed on Western blots. Immunoprecipitated recombinant DoGMP1-GFP fusion proteins were visualized on Western blots with an anti-GFP antibody (product code ab290, Abcam, Cambridge, U.K.) and goat anti-rabbit IgG-HRP (catalog number sc-2301, Santa Cruz Biotechnology, Inc., Santa Cruz, CA, USA).

### Measurement of and mannose content of water-soluble polysaccharides

The above-ground parts (leaves, flowers and stems) from two-month-old *A. thaliana* plants in the reproductive stage were used to determine water-soluble polysaccharide content. Samples were powdered by a DFT-50 pulverizer (Xinno Instrument Equipment Inc., Shanghai, China) after drying in an oven at 85 °C for 6 h. To analyze the mannose content of water-soluble polysaccharides, 0.3 g of powder was pre-extracted with 80% ethanol for 2 h then further extracted with 100 mL of distilled water for 2.5 h at 100 °C. The polysaccharides in the solution were precipitated in 4 volumes of 100% ethanol at 4 °C overnight then centrifuged at 10,000 rpm for 20 min. The residue was re-dissolved in 20 mL of distilled water. The mannose content of the water-soluble polysaccharides was also determined by high performance liquid chromatography (HPLC), as described by He *et al*.^19^.

### Germination assays under salinity stress

Seeds of all genotypes used in germination assays were grown simultaneously, and harvested and stored under similar conditions. Three transgenic lines of T5 homozygote plants and WT plants were surface sterilized by immersion for 10 min in 1% sodium hypochlorite, and then rinsed six times with sterile distilled water. One hundred surface-sterilized seeds of each line were seeded on plates filled with BM supplemented with NaCl, or not. According to the results of a pre-experiment trial, an optimum concentration of 150 mM was used as the NaCl stress treatment. After 2 days of stratification at 4 °C in the dark, the plates were incubated in a 16-h photoperiod (100 μmol m^−2 ^s^−1^) at 22 °C. Germination, which was considered to have occurred if the radicle emerged from the seed coat, was scored daily for 1–7 days. On the seventh day, seedlings were photographed with a Leica S8 APO stereomicroscope (Leica Microsystems Ltd., Heerbrugg, Switzerland). Seeds sown in BM served as the control. Each experiment was performed in three biological replicates.

### Salinity stress treatment for *Arabidopsis thaliana* seedlings

Sterilized seeds were germinated on BM at 22 °C under a 16-h photoperiod (100 μmol m^−2 ^s^−1^) after stratification for 2 days at 4 °C in the dark. Five-day-old seedlings were transferred to fresh BM supplemented with 150 mM NaCl and cultured at 22 °C under a 16-h photoperiod. Root length was measured and photographs were taken after 7 days. Twelve plants were used in each experiment, and all experiments were repeated three times.

### Hydrogen peroxide staining

*A. thaliana* seedlings (12-d old) that were grown on BM were transferred to fresh BM supplemented with 150 mM NaCl, or not, and then cultured at 22 °C under a 16-h photoperiod. Seedlings transferred to BM served as the control. Seedlings were harvested after 48 h and stained with 3,3′-diaminobenzidine (DAB) (D5637, Sigma-Aldrich)^25^. Eight plants of each treatment were used in each analysis, and all experiments were repeated three times.

### Semi-quantitative RT-PCR

One hundred sterilized seeds of transgenic lines #1 and #3, as well as WT were germinated on BM at 22 °C under a 16-h photoperiod (100 μmol m^−2 ^s^−1^). Total RNA was extracted from seven-day-old *A. thaliana* seedlings using TRIzol reagent (Invitrogen, Carlsbad, CA, USA) following the manufacturer’s protocol. Two μg of each RNA sample were reverse transcribed for the first-strand cDNA using M-MLV reverse transcriptase (Promega, Madison, WI, USA) according to the manufacturer’s instructions after treatment with RNase-free DNase (Takara Bio Inc.) to remove any residual genomic DNA. The DreamTaq^TM^ Green PCR Master Mix Kit (Takara Bio Inc.) was used for amplification. The following thermocycling conditions were applied: initial denaturation at 94 °C for 1 min; 30 cycles of 94 °C for 30 s, 55 °C for 30 s and 72 °C for 1 min; final extension at 72 °C for 10 min in a LabCycler Standard Plus PCR system (SENSOQUEST, Hannah, Germany). The amplified products were separated on a 1.5% agarose gel stained with ethidium bromide (EtBr) and photographed in a Bio Sens SC 710 system. The gene-specific primers for *DoGMP1*, which was used in semi-quantitative RT-PCR, and the *A. thaliana* ubiquitin 10 gene (*AtUBQ10*, TAIR accession number: AT4G05320.2), which was used as the control, are listed in Supplementary Table 4. *AtUBQ10* was used as the internal control based on the advice of Zhao *et al*.^26^.

### Quantitative real-time PCR (qRT-PCR) analysis

The cDNAs described above also used for qRT-PCR analysis. The gene-specific primer pairs were designed for qRT-PCR by online Primerquest software (listed in Supplementary Table 4). qRT-PCR was performed using the SYBR Premix Ex Taq^TM^ Kit (Takara Bio Inc.) in an ABI 7500 Real-time system (ABI, CA, USA). Amplification conditions were 95 °C for 2 min, followed by 40 cycles of amplification (95 °C for 15 s, 60 °C for 1 min) and plate reading after each cycle. *AtUBQ10* served as the control based on the recommendation of Zhao *et al*.^26^. The gene-specific primers used for qRT-PCR are listed in Supplementary Table 4.

### Statistical analyses

All data were analyzed using SigmaPlot12.3 software (Systat Software Inc., San Jose, California, USA) using one-way analysis of variance (ANOVA) followed by Dunnett’s test. *P* < 0.05 was considered to be statistically significant.

## Results

### Analysis of three cloned *DoGMP* genes from *D. officinale*

Based on an in-house transcriptome reference database of *D. officinale* sequences, three *GMP* genes were identified. Their full-length cDNAs, which were obtained by RACE, were named *DoGMP1, DoGMP2* and *DoGMP3*. The complete *DoGMP1* cDNA contains 1528 bp with an open reading frame (ORF) of 1086 bp encoding a protein of 361 amino acid residues with a calculated molecular mass of 39.373 kDa. The complete *DoGMP2* cDNA contains 1492 bp with an ORF of 1248 bp encoding a protein of 415 amino acid residues with a calculated molecular mass of 45.875 kDa. *DoGMP3* contains an ORF of 1086 bp encoding a protein of 361 amino acid residues with a calculated molecular mass of 39.604 kDa. The full-length cDNAs of *DoGMP1*-*3* were submitted to GenBank with the following accession numbers: *DoGMP1*, KF195559; *DoGMP2*, KF195560; and *DoGMP3*, KP203853.

**Figure 1:**
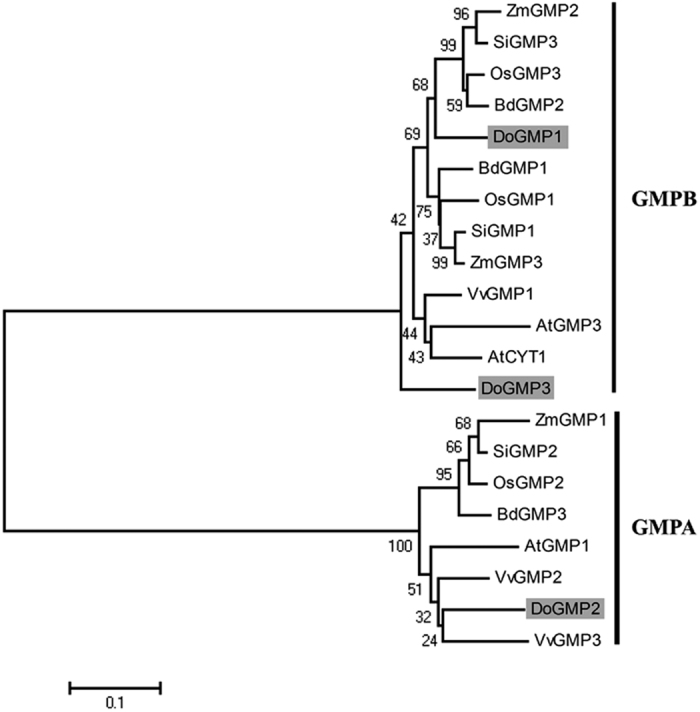
Molecular phylogenetic tree of the amino acid sequences of the GDP-mannose pyrophosphorylase family of higher plants and three DoGMP proteins from *D. officinale*. The tree was constructed using MEGA 4 by the neighbor-joining method. GMP proteins used for alignment are as follows: AtGMP1, NP_177629; AtCYT1, NP_001189713; AtGMP3, NP_191118; VvGMP1, XP_002282422; VvGMP2, XP_002283703.1; VvGMP3, XP_002281959.1; SlGMP1, XP_004240924.1; SlGMP2, XP_004246437.1; SlGMP3, XP_004236149.1; OsGMP1, NP_001044795; OsGMP2, NP_001049332.1; OsGMP3, NP_001049673.1; BdGMP1, XP_003564604.1; BdGMP2, XP_003558261; BdGMP3, XP_003558532.1; ZmGMP1, NP_001131394.1; ZmGMP2, NP_001142215.1; ZmGMP3, NP_001142302.1; SiGMP1, XP_004977599.1; SiGMP2, XP_004985259; SiGMP3, XP_004984923.1; SiGMP4, XP_004970565.1.

Sequence alignment analysis by ClustalX2 showed that all the GMP proteins from *D. officinale, A. thaliana* and *O. sativa* displayed diverse sequence/structure similarity relationships (Supplementary Fig. 1). The conservation of these primary sequences indicated that these proteins had a similar catalytic function. In a further search for conserved domains, GMP proteins were shown to contain four conserved motifs: the pyrophosphorylase signature sequences, a nucleotidyl transferase domain, a metal binding site (D, DxG or D, QxK) and the GMP active site (Supplementary Fig. 1). The conserved VEKP sequence included in the GMP active site is a mannose-1-phosphate binding site of GMP^27^. To examine the relationship between the three DoGMP proteins and other GMP members in plants, a phylogenetic analysis was performed among GMP protein sequences using MEGA 4^28^. These were divided into two groups: GMPA and GMPB. DoGMP1 and DoGMP3 formed part of the GMPB group while DoGMP2 made up the GMPA group (Fig. 1).

### DoGMP1 protein localized in the cytoplasm

**Figure 2:**
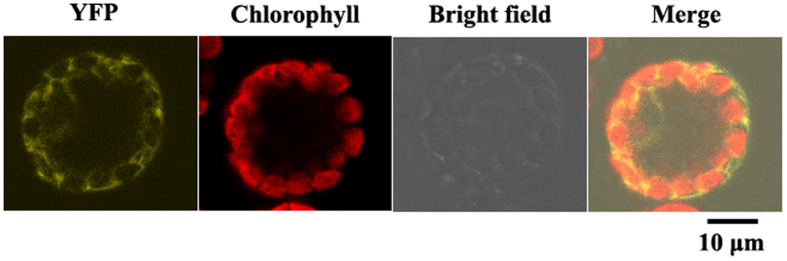
DoGMP1 protein localized in the cytoplasm. (**A**) YFP. (**B**) Autofluorescence of chlorophyll (red). (**C**) Visible light. (**D**) Merged images.

GMP catalyzes the conversion of mannose-1-phosphate to GDP-mannose in the cytoplasm^29^. The OsGMP protein of *O. sativa* is also localized in the cytoplasm^10^. To verify whether DoGMP1 is indeed a cytoplasmic protein, the DoGMP1 protein was fused with YFP to construct a DoGMP1-YFP fusion protein. Transient expression in mesophyll protoplasts of *A. thaliana* seedlings was observed confirming that DoGMP1 protein is indeed a cytoplasmic protein (Fig. 2).

### Analysis of stress-related *cis-*regulatory elements in the putative promoters of *DoGMP*s

**Figure 3:**
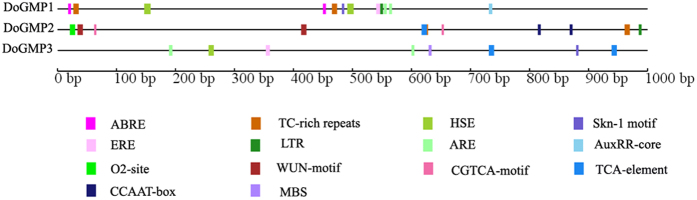
Analysis of the important *cis-*regulatory elements in the 1 Kb upstream sequences of *DoGMP*s. ABRE, a *cis*-acting element involved in the abscisic acid responsiveness; ARE, a *cis*-acting regulatory element essential for anaerobic induction; AuxRR-core, a *cis*-acting regulatory element involved in auxin responsiveness; ERE, an ethylene-responsive element; HSE, a *cis*-acting element involved in heat stress responsiveness; LTR, a *cis*-acting element involved in low-temperature responsiveness; Skn-1_motif, a *cis*-acting regulatory element required for endosperm expression; TC-rich repeats, a *cis*-acting element involved in defense and stress responsiveness; CCAAT-box, a MYBHv1 binding site; CGTCA-motif, a *cis*-acting regulatory element involved in MeJA responsiveness; O_2_-site, a *cis*-acting regulatory element involved in zein metabolism regulation; WUN-motif, a wound-responsive element; MBS, a MYB-binding site involved in drought induction.

Studies have demonstrated that *GMP* family members are involved in tolerance to abiotic stresses^10^. To understand the possible stress-related *cis-*regulatory elements in the promoter regions of *DoGMPs*, about 1200, 1000 and 1800 bp of the translation start site of *DoGMP1, DoGMP2* and *DoGMP3* were obtained namely, respectively. 1 kb of the promoter sequences of each gene was used to predict the stress-related *cis-*regulatory elements (Supplementary Text 1–3). A low-temperature responsiveness element and defense and stress responsiveness elements were found both in *DoGMP1* and in *DoGMP2* (Fig. 3). The HSE element, which is involved in heat stress responsiveness, was found both in *DoGMP1* and in *DoGMP3* (Fig. 3). In addition, the three *DoGMP*s contained several plant hormone responsiveness elements such as an ethylene responsive element, a salicylic acid (SA) inducible element, a methyl jasmonate (MeJA) inducible element and an abscisic acid (ABA) responsive element (Fig. 3). Plant hormones such as ethylene, SA, MeJA and ABA play important roles during a plant’s response to abiotic stresses^30^,^31^,^32^,^33^. These results suggest the putative roles of these three *DoGMP*s in the response of *D. officinale* to abiotic stresses.

### Mannose content of water-soluble polysaccharides increased in *35S*:*DoGMP1* transgenic lines

**Figure 4:**
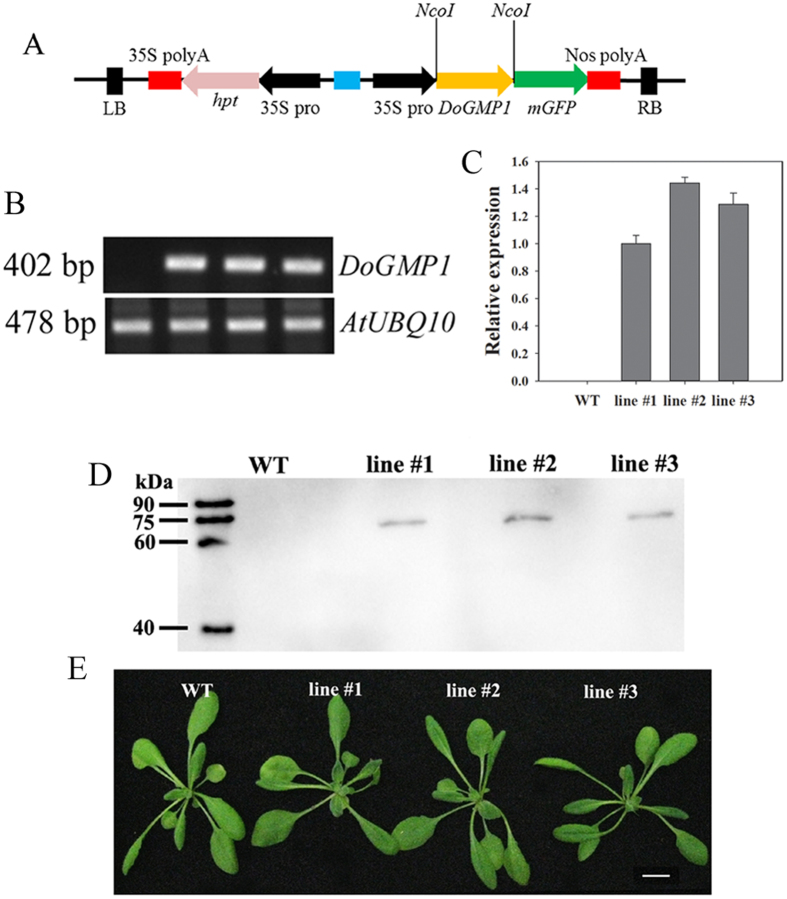
Overexpression of the *DoGMP1* gene in *Arabidopsis thaliana*. (**A**) Schematic presentation of the *35S*:*DoGMP1* overexpression vector. (**B**) Analysis of the *DoGMP1* gene in WT and transgenic lines by semi-quantified PCR. Total RNA was isolated from 7-day-old WT and homozygous *35S*:*DoGMP1* transgenic *A. thaliana* transgenic lines under control conditions. (**C**) Analysis of the *DoGMP1* gene in WT and transgenic lines by qPCR analysis. Total RNA was isolated from 7-day-old WT and s *35S*:*DoGMP1* transgenic lines under control conditions. Expression levels in transgenic lines were calculated relative to transgenic line #1, which exhibited the lowest transgene expression. (**D**) Analysis of the DoGMP1-GFP fusion protein expression in WT and transgenic lines by Western blotting. (**E**) Seedlings of WT and transgenic lines about one month old showed no obvious phenotypic changes. WT, wild-type plant; *35S*:*DoGMP1* transgenic lines: line #1, line #2 and line #3. Bar = 1 cm.

The *vtc*-*1* (known as *cyt1*) mutant of *A. thaliana* is hypersensitive to abiotic stresses such as ozone stress, sulfur dioxide, ultraviolet B irradiation and salt stress^8^,^34^. This indicates that *AtCYT1* plays an important role in improving the abiotic stress tolerance of plants. Comparison of the *GMP* protein sequences in *A. thaliana* and *D. officinale* revealed that *DoGMP1* was most similar to *AtCYT1* and may thus play a similar role. Therefore, to analyze the functional role of the *DoGMP1* gene, transgenic *A. thaliana* plants that constitutively overexpressed the *DoGMP1* gene driven by the CaMV *35S* promoter (*35S*:*DoGMP1-GFP*) were generated (Fig. 4A). Semi-quantitative RT-PCR and qRT-PCR were used to determine *DoGMP1* gene expression in transgenic plants. The *DoGMP1* gene could not be detected in WT plants but was detected in all three transgenic lines (Fig. 4B,C). Western blot analyses confirmed the expression of DoGMP1-GFP fusion proteins of correct size in all of the transgenic lines (Fig. 4D). No obvious phenotypic changes were observed among WT and transgenic plants when cultured in soil (Fig. 4E).

**Figure 5:**
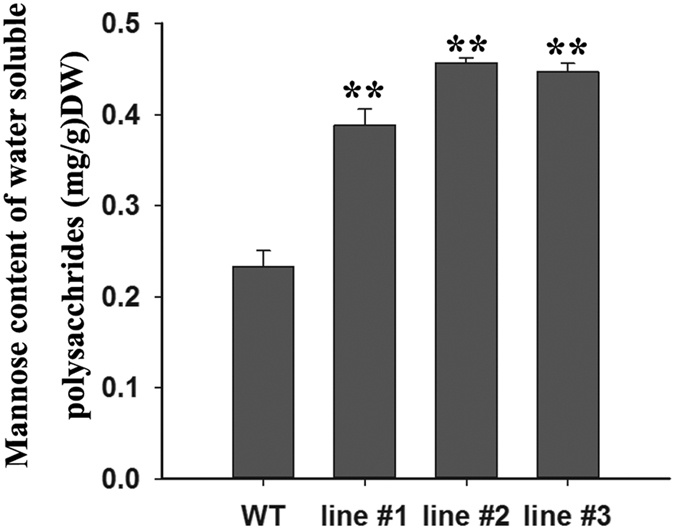
Analysis of mannose content of *35S*:*DoGMP1* transgenic lines. Each data bar represents the mean ± standard deviations (SD) (n = 3). Asterisks indicate significant differences between *35S*:*DoGMP1* transgenic lines and WT. **, indicates P < 0.01 between WT and transgenic lines by ANOVA/Dunnett’s test. WT, wild-type; *35S*:*DoGMP1* transgenic lines: line #1, line #2 and line #3. DW, dry weight.

**Figure 6:**
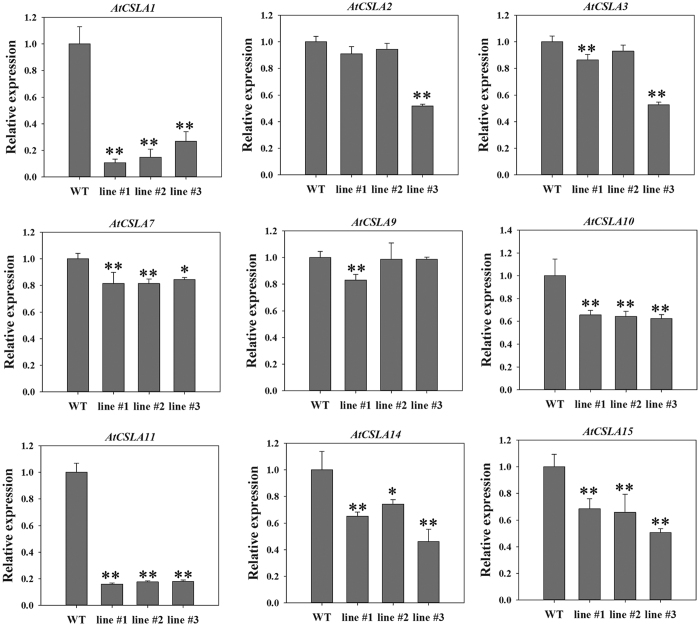
qRT-PCR was used to analyze the expression of *AtCSLA* genes involved in polysaccharide synthesis from one-week-old transgenic lines and WT plants. Transcripts were normalized to actin gene (*AtUBQ10*) expression. Asterisks indicate significant differences between *35S*:*DoGMP1* transgenic lines and WT. *, indicates P < 0.05, **, indicates P < 0.01 between WT and transgenic lines by ANOVA/Dunnett’s test. Data are means and SD from three biological replicates each with 100 seeds. WT, wild-type; *35S*:*DoGMP1* transgenic lines: line #1, line #2 and line #3.

GDP-mannose, the product of the GMP enzyme, is the mannose donor for the synthesis of mannose-containing polysaccharides. To gain insight into the mannose content of these extracted polysaccharides, mannose content was determined by HPLC, which showed that mannose content in the transgenic lines increased significantly more than in the WT plant (Fig. 5). Over-expression of the *DoGMP1* gene resulted in an obvious increase in the content of mannose in *A. thaliana* plants, suggesting that the *DoGMP1* gene is involved in the biosynthesis of water-soluble polysaccharides, which are mannose-containing polysaccharides. Previous studies demonstrated that the backbone of mannan synthase, which is encoded by *CSLA* family members, is made by using GDP-mannose as the donor^15^,^35^. The *CSLA* family belongs to the *CesA* superfamily and includes nine *CSLA* members in *A. thaliana*: *AtCSLA1, AtCSLA2, AtCSLA3, AtCSLA7, AtCSLA9, AtCSLA10, AtCSLA11, AtCSLA14* and *AtCSLA15*^36^. Since the mannose content increased significantly when the *DoGMP1* gene was overexpressed in *A. thaliana*, the relative expression level of these nine *AtCSLA* genes were analyzed. *AtCSLA1, AtCSLA7, AtCSLA10, AtCSLA11, AtCSLA14* and *AtCSLA15* were significantly down-regulated in all *35S*:*DoGMP1* transgenic lines (Fig. 6), which indicates that the *DoGMP1* gene has a negative role in the transcriptional regulation of *AtCSLA* genes.

### Germination of *35S*:*DoGMP1* transgenic lines improved under salinity stress

**Figure 7:**
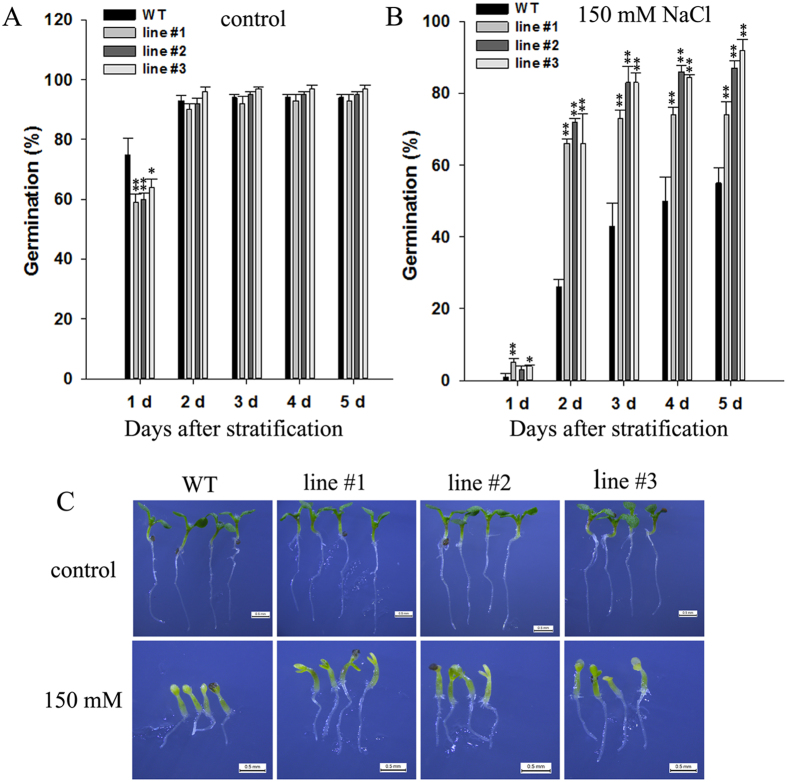
Analysis of the germination of *35S*:*DoGMP1* transgenic seeds under salinity stress. Seeds of WT and transgenic lines were cultured on BM (**A**) without salt or (**B**) with 150 mM NaCl. (**C**) Seedlings on the seventh day after stratification from A and B. Asterisks indicate significant differences between *35S*:*DoGMP1* transgenic lines and WT. *, indicates P < 0.05, **, indicates P < 0.01 between WT and transgenic lines by ANOVA/Dunnett’s test. Each data bar represents the mean ± SD of 100 seeds. Bar = 0.5 mm. WT, wild-type; three *35S*:*DoGMP1* transgenic lines: line #1, line #2 and line #3.

Salinity, a critical factor influencing seed germination^37^, reduces germination rate and delays seed emergence^38^,^39^. Soluble sugars or polysaccharides may play important roles in the mechanisms of salt defense^40^,^41^. To investigate the effect of salinity stress on the germination of seeds from WT and transgenic lines, seeds were germinated on BM containing 150 mM NaCl (or not) to explore germination ability under salt stress. In the control group, seeds from all three transgenic lines germinated later than WT seeds one day after stratification, although seeds from WT and transgenic lines showed no obvious differences in the following days when germination was scored (Fig. 7A). In contrast, a significant difference in germination was observed between *35S*:*DoGMP1* lines and WT when MS medium was supplemented with 150 mM NaCl. The three 35S:*DoGMP1* transgenic lines exhibited a higher germination percentage than WT seeds on all scoring days (Fig. 7B). In particular, two days after stratification on 150 mM NaCl there was a significant difference in the germination percentage of *35S*:*DoGMP1* lines #1, #2 and #3 (65.7%, 72.0%, and 69.0%, respectively) compared with 26.0% for WT seeds (Fig. 7B). The seedlings of both WT and the three transgenic lines showed a similar phenotype when germinated on half-strength MS medium without NaCl (Fig. 7C). However, roots of transgenic lines were longer than the roots of WT seedlings and seedling growth was more vigorous in transgenic lines than WT seedlings when seeds were germinated under salinity stress at 7 days after stratification (Fig. 7C). This implies that *DoGMP1* plays a role as a positive regulator in seed germination of *A. thaliana* under salt stress.

### *35S*:*DoGMP1* transgenic lines grew better than WT plants under salinity stress

**Figure 8:**
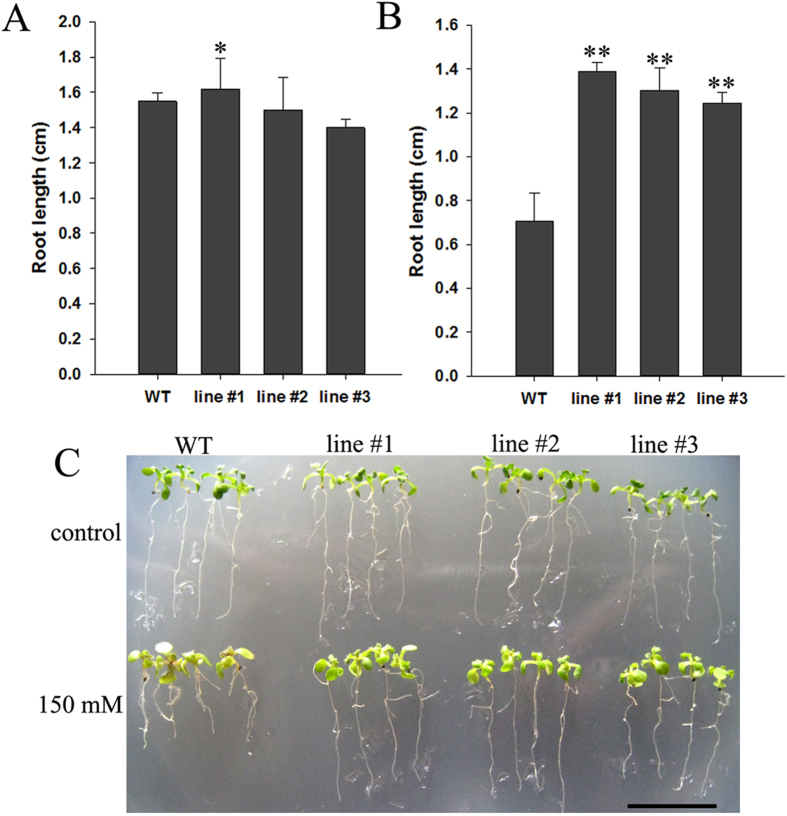
Analysis of the growth of *35S*:*DoGMP1* transgenic plants under salinity stress. Seeds of WT and transgenic lines were cultured on half-strength MS medium (**A**) without salt or (**B**) with 150 mM NaCl. (**C**) Seedlings cultured on the 7^th^ day after five-day-old seedlings were transplanted to half-strength MS containing 150 mM NaCl, or not. Each data bar represents the mean ± SD of 12 plants. Asterisks indicate significant differences between *35S*:*DoGMP1* transgenic lines and WT. *, indicates P < 0.05, **, indicates P < 0.01 between WT and transgenic lines by ANOVA/Dunnett’s test. Bar = 1 cm. WT, wild-type; three *35S*:*DoGMP1* transgenic lines: line #1, line #2 and line #3.

To analyze the growth of transgenic seedlings under salinity stress, five-day-old seedlings of *35S*:*DoGMP1* transgenic *A. thaliana* versus WT plants were grown on BM containing 150 mM NaCl (or not) to further characterize the response of the over-expressing transgenic plants to abiotic stress at the seedling stage. When the seedlings of WT and transgenic lines were grown under control conditions, no obvious differences were observed in root length (Fig. 8A,C). However, in the presence of 150 mM NaCl, the roots of WT plants were markedly shorter than those of transgenic lines (Fig. 8B,C). The root length of WT seedlings was about 0.75 cm, while that of transgenic lines #1, #2 and #3 was 1.39, 1.33, and 1.24 cm, respectively. The salinity stress test showed that transgenic seedlings could grow better than WT seedlings under salinity stress. These results suggest that *DoGMP1* is able to provide *A. thaliana* seedlings with tolerance to salinity stress.

**Figure 9:**
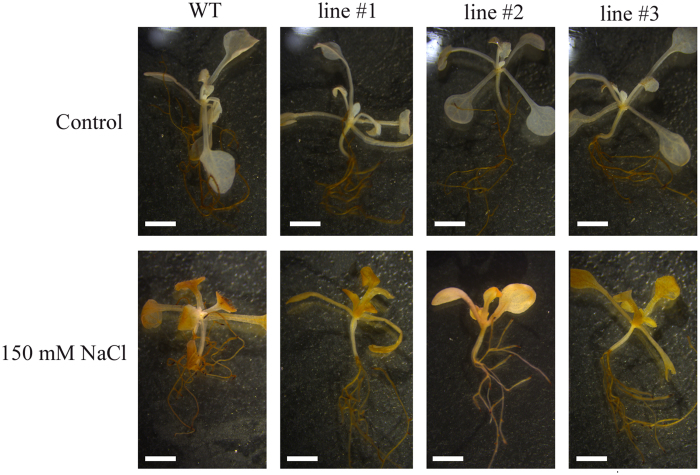
Detection of hydrogen peroxide (H_2_O_2_) by DAB staining. H_2_O_2_ was detected in 12-day-old WT and *35S*:*DoGMP1* transgenic lines grown in BM and treated with 150 mM NaCl for 48 h. Brown regions indicate the H_2_O_2_ level. Bar = 1 mm. WT, wild-type; three *35S*:*DoGMP1* transgenic lines: line #1, line #2 and line #3.

To investigate whether the better growth of transgenic lines relative to WT was associated with accelerated ROS accumulation, DAB staining was carried out in WT and transgenic lines after control and salt stress treatment. No coloration was observed on the leaves when the seedlings of WT and transgenic lines were cultured in BM while the roots were slightly stained (Fig. 9A). In contrast, the leaves and roots from both WT and transgenic lines under salt stress were strongly stained relative to the control (Fig. 9B). The WT seedlings contained a higher level of H_2_O_2_ in leaves and root tips than seedlings of transgenic lines when cultured in BM supplemented with 150 mM NaCl (Fig. 9B).

## Discussion

GMP catalyzes the reaction from mannose-1-phosphate to GDP-mannose and uses cofactor Mg^2+^ as a key switch for effective and continuous enzyme production^42^,^43^. Two types of *GMP* genes, namely *GDP*-*mannose pyrophosphorylase A* (*GMPA*) and *GDP*-*mannose pyrophosphorylase B* (*GMPB*), were found in fungus (*Saccharomyces cerevisiae*)^44^,^45^, pig (*Sus scrofa*)^46^, humans (*Homo sapiens*)^29^ and a higher plant, rice^10^. The protein sequences of GMPA and GMPB are very similar, but those of GMPA members are generally longer than those of GMPB members. Three *DoGMP* genes cloned from *D. officinale* showed high similarity with *GMP* sequences of other plant species (Supplementary Fig. 1). DoGMP1 and DoGMP3 belong to the GMPB group while DoGMP2 is a member of the GMPA group (Fig. 1). These three DoGMP proteins are likely to have the same catalytic function similar to other GMP proteins. DoGMP1 was localized in the cytoplasm, as was observed in other species such as *Leishmania parasites, Homo sapiens* and *O. sativa*^10^,^47^,^48^.

Studies have demonstrated that GDP-mannose is the active nucleotide sugar that provides mannose for the biosynthesis of mannan polysaccharides by mannosyltransferase^49^. Transgenic potato plants whose GMP was inhibited by an antisense construct, showed 30–50% less mannose content than WT level^4^. In the present study, the *DoGMP1* gene contributed to the mannose content of water-soluble polysaccharide, consistent with previous studies. This indicates that the *DoGMP1* gene plays an important role in mannose-containing polysaccharide synthesis in *D. officinale*. Feedback regulation is very common in plant metabolism to maintain a balance. For example, carbon metabolites have feedback regulation during photosynthesis^50^ while amino acids play a role in the feedback regulation of amino acid biosynthetic pathways^51^. Six genes (*AtCSLA1, AtCSLA7, AtCSLA10, AtCSLA11, AtCSLA14* and *AtCSLA15*) related to mannose-containing polysaccharide synthesis were down-regulated in all of transgenic *A. thaliana* lines (Fig. 6). The *AtCSLA* genes were down-regulated significantly when BM was supplemented with 10 mM exogenous mannose (Supplementary Fig. 2). This suggests that high mannose-containing polysaccharide content has a negative feedback regulation of upstream genes.

Salinity stress has negative effects on seed germination and plant growth, and osmotic stress decreases water potential, consequently restricting water uptake by dry seeds and roots^52^,^53^. Water uptake during seed germination can be divided into three phases: Phase I, the initial absorption of water (imbibition), is primarily a physical process; Phase II, a plateau with stable water content; Phase III, water uptake by protrusion of the radicle^54^,^55^. At the imbibition stage, proteins and polysaccharides are involved in the absorption of water by the dry seed^56^. As salinity stress builds up, water potential declines, limiting the germination of seeds by negative water potentials. Low molecular weight sugars such as monosaccharides, disaccharides and oligosaccharide serve as an important compatible solute to assist plants in dealing with osmotic stress caused by salinity stress^57^. Water-soluble polysaccharides may serve as a hydrophilic substance and help dry seeds to initially absorb water. The content of low molecular weight sugars, which were extracted by hot 80% ethanol, in the mature seeds of *35S*:*DoGMP1* transgenic lines #1–3 was 54.3, 59.9 and 58.6 mg g^−1^ DW, respectively, while that of WT was 42.3 mg·g^−1^ DW (Supplementary Fig. 3A). The content of water-soluble polysaccharides in the mature seeds of the three transgenic lines was also significantly higher (35.6, 40.4 and 37.7 mg·g^−1^ DW, respectively) than that of WT plants (31.6 mg·g^−1^ DW, Supplementary Fig. 3B). The mature seeds of *35S*:*DoGMP1* transgenic lines had a higher content of low molecular weight sugars and water-soluble polysaccharides than WT plants. This may help transgenic seeds absorb water from a saline environment at the imbibition phase. Thus, the germination of seeds of transgenic lines was higher than of WT seeds.

Salinity stress not only intervenes in seed germination, but also influences seedling establishment. The decrease in water potential caused by salinity stress results in reduced cell growth, root growth and shoot growth^53^. The root growth of WT plants was distinctly inhibited by salinity stress (Figs 7C and 8). Salt stress reduces the ability of plants to take water up from the soil^58^,^59^. To deal with water stress, plants accumulate compatible solutes such as proline and sugars to reduce the cell water potential and absorb water from soil^60^. The roots of transgenic plants contained higher levels of low molecular weight sugars and water-soluble polysaccharides than WT plants (Supplementary Fig. 4), which can be used as compatible solutes to reduce cell water potential allowing water to be absorbed from a saline environment. Furthermore, excessive radical oxygen species (ROS) are produced when plants undergo various environmental stresses such as drought, chilling, metal toxicity, UV-B radiation and salinity^61^. *GMP*s are involved in the synthesis of AsA, which acts as a non-enzymatic antioxidant playing important roles in plants’ responses to stresses, and serves to eliminate excess ROS in plant cells^11^,^62^,^63^. The AsA content increased significantly in the three transgenic lines (5.31, 5.37 and 6.71 μmol·g^−1^ FW, respectively; Supplementary Fig. 4) compared to the WT plant (3.70 μmol·g^−1^ FW). This is likely to have increased ROS scavenging. In *DoGMP1*-overexpressing plants, H_2_O_2_, one form of ROS, was found at low levels than in WT plants under salt stress, as revealed by DAB staining (Fig. 9). These results suggest that *DoGMP1* may enhance salt stress tolerance through the up-regulation of water-soluble sugars and AsA, thereby reducing ROS. In contrast, WT plants are unable to mediate the equilibrium of ROS production and scavenging, making the uptake of water from a saline medium very difficult, and subsequently suffering from oxidative damage and water stress, eventually developing a stressed phenotype.

In conclusion, three *DoGMP* genes were cloned from an important traditional Chinese herb, *D. officinale*, which contains abundant mannose-containing polysaccharides. The *DoGMP1* protein located in the cytoplasm catalyzed the synthesis of GDP-mannose. These results provide evidence for the involvement of the *DoGMP1* gene in the biosynthesis of mannose-containing polysaccharides, paving the way for studies on the biosynthesis of bioactive polysaccharides in *D. officinale. 35*:*DoGMP1* transgenic plants showed higher germination and grew better than WT plants under salinity stress. This indicates that the *GMP* gene can be utilized as a candidate gene for improving abiotic stress tolerance in plants.

## Additional Information

**How to cite this article**: He, C. *et al*. *DoGMP1* from *Dendrobium officinale* contributes to mannose content of water-soluble polysaccharides and plays a role in salt stress response. *Sci. Rep.* **7**, 41010; doi: 10.1038/srep41010 (2017).

**Publisher's note:** Springer Nature remains neutral with regard to jurisdictional claims in published maps and institutional affiliations.

## Supplementary Material

Supplementary Information
