# Supplementary material for: DoGMP1 from Dendrobium officinale contributes to mannose content of water-soluble polysaccharides and plays a role in salt stress response
Source: Sci Rep. 2017 Feb 8;7:41010. doi: 10.1038/srep41010 (PMC5296857; doi:10.1038/srep41010)
Supplement: Supplementary Information [file srep41010-s1.doc]

***DoGMP1* from *Dendrobium officinale*** **contributes to mannose content of water-soluble polysaccharides and plays a role** **in salt stress response**

**Chunmei He^1†^** **∙ Zhenming Yu^1, 4†^ ∙** **Jaime A.** **Teixeira da Silva^2^ ∙ Jianxia Zhang^1^ ∙ Xuncheng Liu^1^ ∙ Xiaojuan Wang^1^** **∙ Xinhua Zhang^1^ ∙ Songjun Zeng^1^ ∙ Kunlin Wu^1^ ∙ Jianwen Tan^1^ ∙ Guohua Ma^1^ ∙** **Jianping Luo^3^ ∙ Jun Duan^1*^**

^1^ *Key Laboratory of South China Agricultural Plant Molecular Analysis and Gene Improvement,* *South China Botanical Garden, Chinese Academy of Sciences, Guangzhou 510650, China;*

^2^ *P. O. Box 7, Miki-cho post office, Ikenobe 3011-2,* *Kagawa-ken, 761-0799, Japan;*

^3^ *School of Food Engineering and Biotechnology, Hefei University of Technology, Hefei 230009, China;*

*^4^University of Chinese Academy of Sciences, Beijing 100049, China;*

**^†^** Equal contributors

^*^ Corresponding author: Tel: +86-20-37252993; fax: +86-20-37252978;

*E-mail address:* [duanj@scib.ac.cn](mailto:duanj@scib.ac.cn) (Duan J.)

Methods

Effects of supplemental mannose on *AtCSLA* genes expression

Seven-day-old WT seedlings germinated on BM at 22 °C under a 16-h photoperiod (100 µmol m^-2^ s^-1^) were transferred to fresh BM supplemented with 1 mM or 10 mM of mannose, and cultured at 22 °C under a 16-h photoperiod. Seedlings transferred to fresh BM without mannose served as the control. Seedlings were harvested after treatment for 36 h, then immediately ground in liquid nitrogen with a mortar and pestle. Total RNA was extracted using TRIzol reagent. Two µg of each total RNA sample were reverse transcribed using M-MLV reverse transcriptase (Promega, Madison, WI, USA) and quantitative real-time PCR (qRT-PCR) analysis was performed. Thirty plants were used in each treatment, and all experiments were repeated three times.

Analysis of total AsA

Healthy one-week-old *A. thaliana* seedlings (50 mg), grown on half-strength MS (containing 2% sucrose and 1.2% agar; pH 5.7), were used to detect total AsA content. The seedlings were harvested and kept in liquid nitrogen. Samples were ground in liquid nitrogen and added to a 2 mL tube. One mL of 6% trichloroacetic acid (TCA) was transferred to the tube. Another 1 mL of 6% TCA was used to wash the mortar and pestle and collected in the same tube, which was centrifuged at 4 °C and 12,000 rpm for 20 min to generate an extracted solution. The ascorbate oxidase method used to assay total AsA content was as described by Ueda et al.^1^ Total AsA was calculated as the sum of reduced AsA and oxidized AsA. Each sample was assayed as three replicates.

Analysis of low molecular weight sugars and water-soluble polysaccharides

For seed analysis, whole mature and dry seeds were frozen in liquid nitrogen and grounded to a fine powder using a mortar and pestle, then dried to constant weight in an oven at 80 °C. Ten mg of each powder was pre-extracted twice with 1 mL of hot 80% (v/v) ethanol for 20 min and centrifuged at 9,000 rpm for 10 min. Ethanol (2 mL) was pooled for the analysis of monosaccharides, disaccharides) and oligosaccharides (low molecular sugars) by using the phenol-sulfuric acid method^2^. The pellet was suspended in 2 mL of distilled water, then incubated in an ultrasonic bath for 2 h at 60 °C^3^. After centrifugation at 9,000 rpm for 10 min, the supernatant was collected and used for the analysis of water-soluble polysaccharides by the phenol-sulfuric acid method^2^.

For root analysis, whole roots were excised, cleaned with distilled water, immersed in liquid nitrogen and homogenized to a fine powder by using a mortar and pestle. The powder of each sample was oven-dried at 37 °C until constant weight. Pre-extraction and extraction were performed as described above, and low molecular weight sugars and water-soluble polysaccharides were analyzed by the phenol-sulfuric acid method^2^.

References

1 Ueda, Y., Wu, L. & Frei, M. A critical comparison of two high-throughput ascorbate analyses methods for plant samples. *Plant Physiology and Biochemistry* 70, 418-423 (2013).

2 Dubois, M., Gilles, K. A., Hamilton, J. K., Rebers, P. & Smith, F. Colorimetric method for determination of sugars and related substances. *Analytical Chemistry* 28, 350-356 (1956).

3 Ye, Y. Study on the extract of polysaccharides from *Dendrobium officinale* with ultrasonic method *Journal of Chinese Medicinal Materials* 32, 617-620 (2009).

**
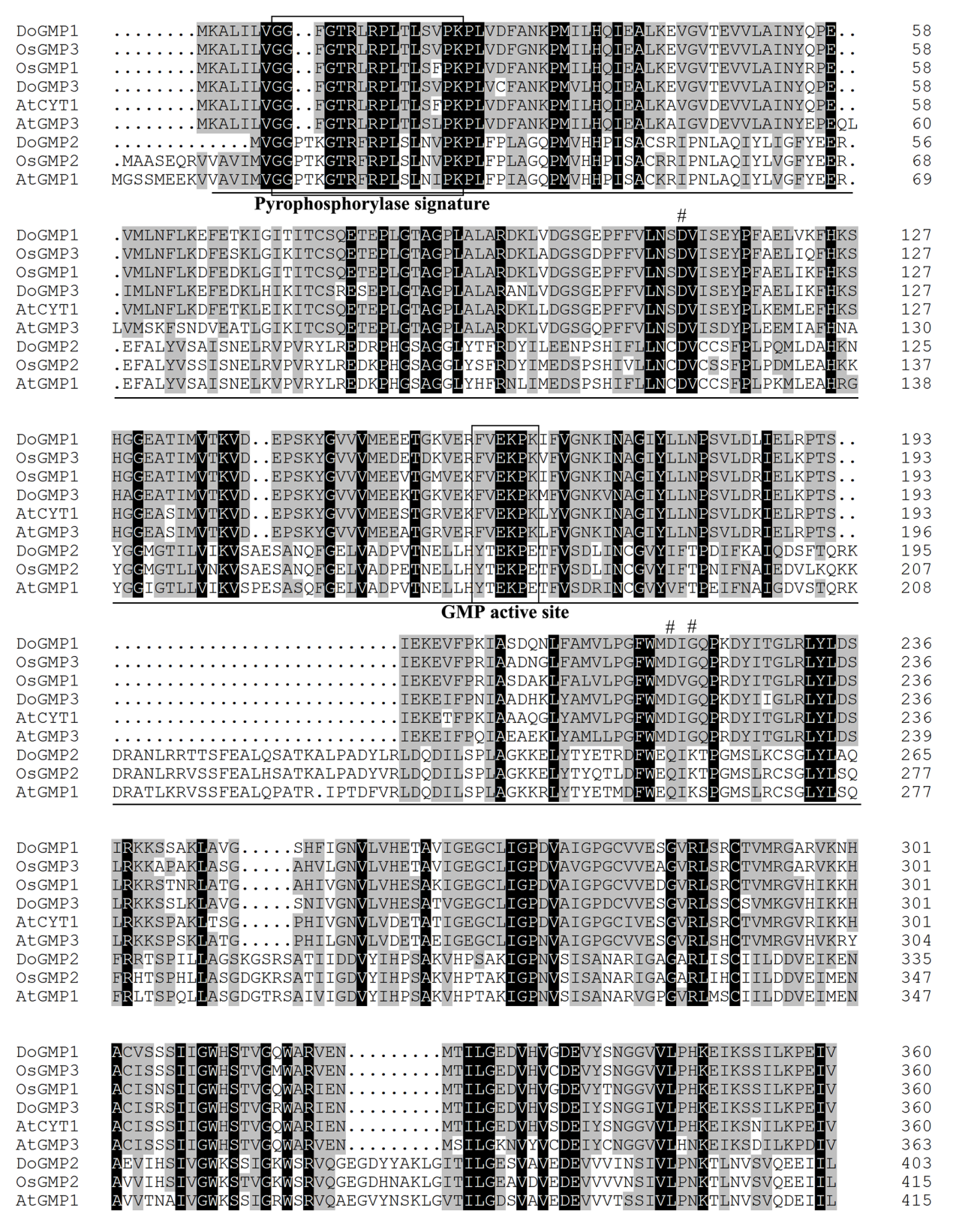
**

**Supplementary Fig. 1** Sequence alignment of the three DoGMP proteins from *D*. *officinale*, AtGMP proteins from *Arabidopsis thaliana* and OsGMP proteins from *Oryza sativa* L. by ClustalX2. Identical residues in the same sequences are shaded black, while similar residues are shaded in gray. The proteins used for sequence alignment are as follows: AtGMP1, NP_177629; AtCYT1, NP_001189713; AtGMP3, NP_191118; OsGMP1, NP_001044795; OsGMP2, NP_001049332.1; OsGMP3, NP_001049673.1. The underlined sequences indicate the nucleotidyl transferase domain. The # indicates a metal-binding site.


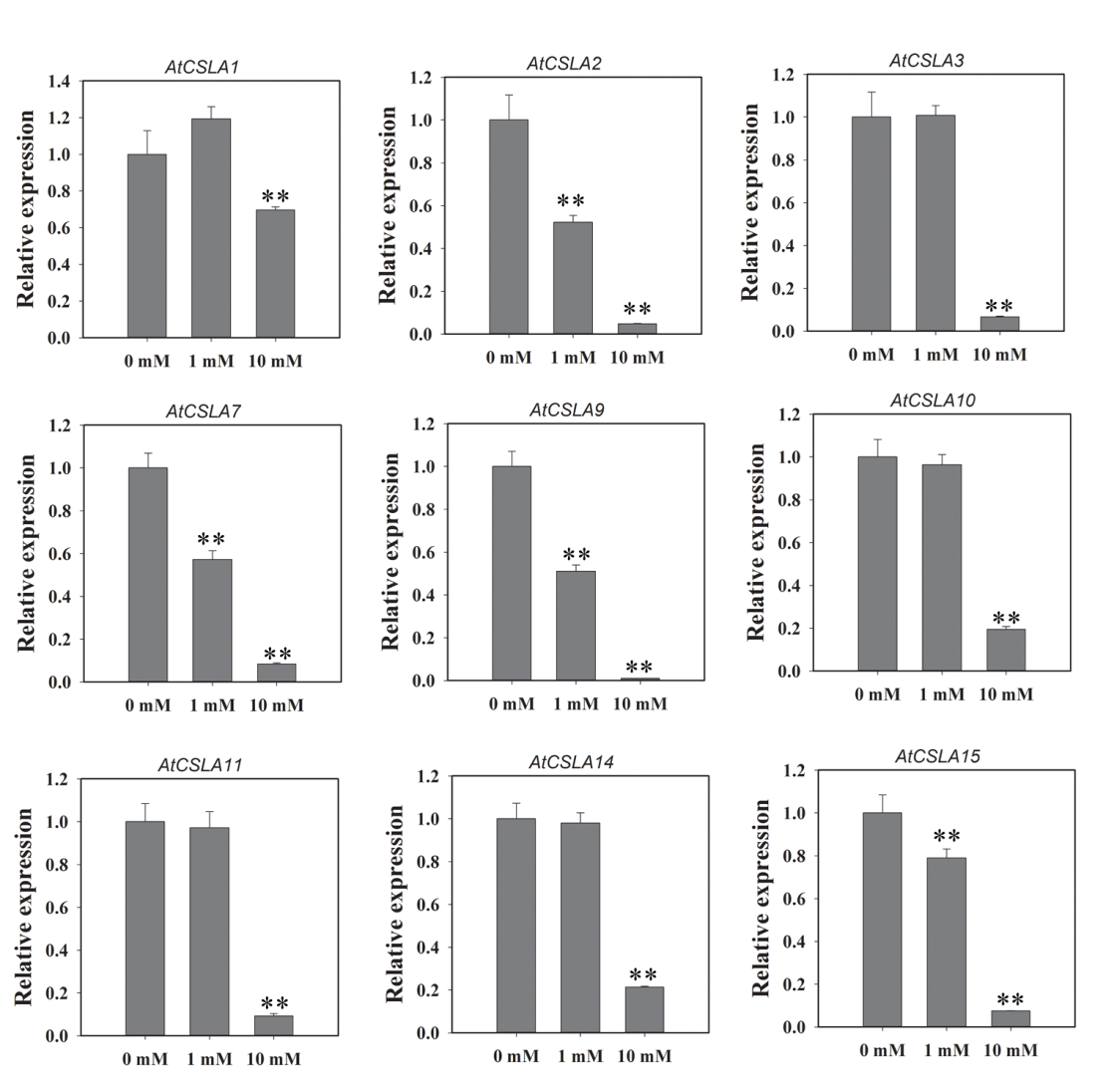


**Supplementary Fig. 2** qRT-PCR was used to analyze the expression of *AtCSLA* genes in WT plants. Transcripts were normalized to actin gene (*AtUBQ10*) expression. The seedlings grew on SB without mannose as the control. Asterisks indicate significant differences between the control and treatment. *, indicates P < 0.05, **, indicates P < 0.01 between the control and treatment by ANOVA/Dunnett's test. Each data bar represents the mean ± SD of 30 seedlings.


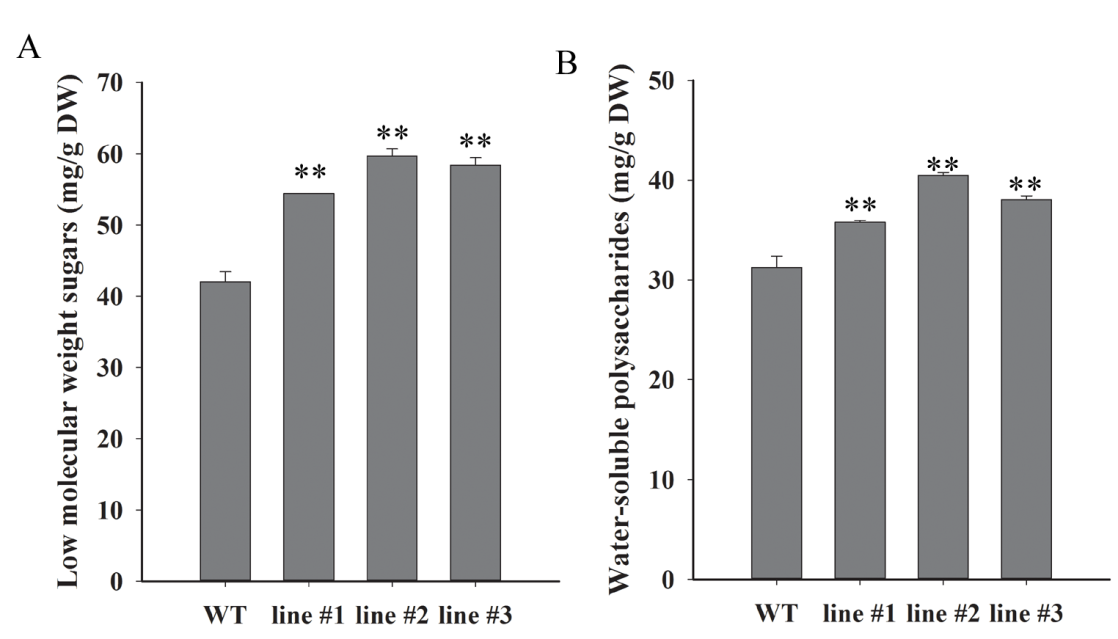


**Supplementary Fig. 3** Analysis of low molecular weight sugars and water-soluble polysaccharides in the seeds of WT and *35S*:*DoGMP1* transgenic lines. DW, dry weight; WT, wild-type; *35S*:*DoGMP1* transgenic lines: line #1, line #2 and line #3. Asterisks indicate significant differences between *35S*:*DoGMP1* transgenic lines and WT. *, indicates P < 0.05, **, indicates P < 0.01 between WT and transgenic lines by ANOVA/Dunnett's test. Each data bar represents the mean ± SD (n = 3).


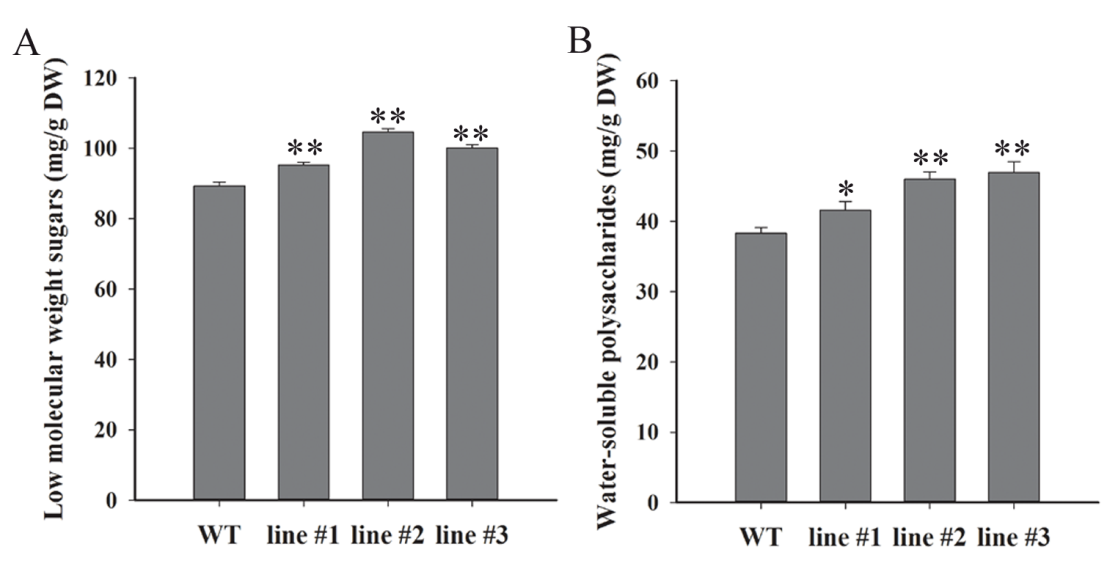


**Supplementary Fig. 4** Analysis of low molecular weight sugars and water-soluble polysaccharides in the roots of WT and *35S*:*DoGMP1* transgenic lines. The roots were harvested from 3-week-old plantlets of WT and *35S*:*DoGMP1* transgenic lines that were grown on BM under control conditions. DW, dry weight; WT, wild-type; *35S*:*DoGMP1* transgenic lines: line #1, line #2 and line #3. Asterisks indicate significant differences between *35S*:*DoGMP1* transgenic lines and WT. *, indicates P < 0.05, **, indicates P < 0.01 between WT and transgenic lines by ANOVA/Dunnett's test. Each data bar represents the mean ± SD (n = 3).

**
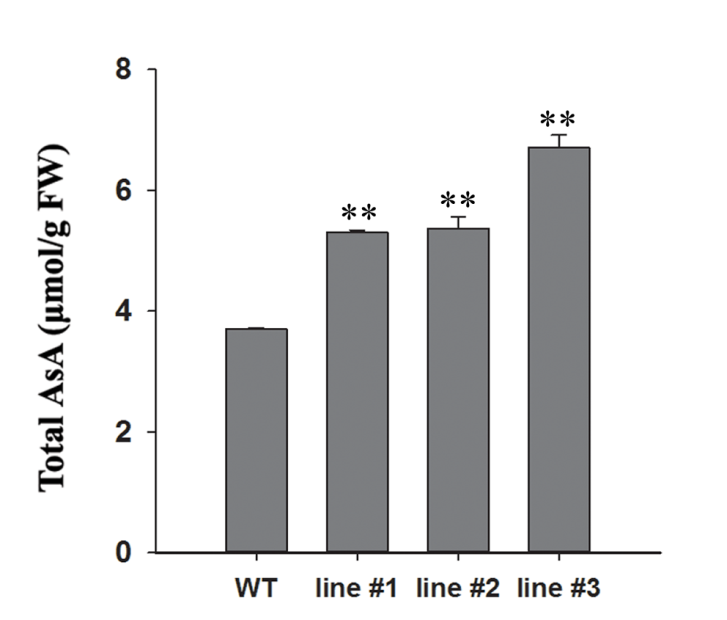
**

**Supplementary Fig. 5** Analysis of total AsA content of *35S*:*DoGMP1* transgenic lines. FW, fresh weight; WT, wild-type; *35S*:*DoGMP1* transgenic lines: line #1, line #2 and line #3. Asterisks indicate significant differences between *35S*:*DoGMP1* transgenic lines and WT. *, indicates P < 0.05, **, indicates P < 0.01 between WT and transgenic lines by ANOVA/Dunnett's test. Each data bar represents the mean ± SD (n = 3).

**Supplementary Text 1 *DoGMP1* putative promoter sequence:**

5′-TTAAATATATAAATTTTCTACGTGTAAATTCTCTAAAATAAATTGTTTAGGAAGCTTCCAAACTGGCGGGTGTATATAGCCGAACAGATCGTTCAACACGGACTTGCGCAAGGTAAACGAGCAAGGAGCCGTTAATAATCGGGTTCTCGCATTTTCTCTCCGCGTCTCCTTCTTGCTCAGCGCACGAAGGTAGTAACGGGAGAGAATAGATCGAATATCTTCGGATTTCCCGCCCTCCATCTCGAATCTGACCTCTAATACGGTATTGGCCGTTCAAATCGACCAGTTTTTTTTTCTTCTTTTCCAAATTTCTGTAATTCAATTTTCATTTTAGTGTTTCTCCCTAGATCTGCATCAATTCCTTTCTTAGGGGTTTTATATCTTCTTTTATCGTCCATGGATCTCATGCAATGTTGCGGGATCTGGATGCTTTGGCGTTTTGATTGGGGCTACGTGCTTCAATTCATTTTCTTTATTTTGCAGTCATTTGCGCAATTTTTTAGAAGGCCAACGCATTTGGCCTTTGCTGTTCCGAGATTGTTTGAAATTTCGGTGGTTTTCTTGGTTTGTTGTTGGAAATATTCAGATCCGACGTGTGATTCGCTCTTTAGTTTTGTGAATGAATGTTAAAGAGTAAAGCTAGGCATCTTAAAAATATCTCTGTTATTCCATAAATTAGCCCTTCAAACAATCCAATATCAGTTCTAATTTAGAAAGGAATGGTAAAAAATGGTCCATCTTGGTCTCTAGTCTTTCTTTGCTACGAGATTTTGTGTTCATAGATGATCTATCTCTTGTTTTAGCTTGAAATCTTTATTGTGGCTCTAGCCAAAGCAATTCTAGCTTTTTTTGCGGCCTGATCCTTATCCCTTTTAGCCATTCTTCTTGGCGGAGCTCCAGAGCTGGAGACCCGGCTGTTGAAGCTCAGAACTTTCTTAGATTCCTTCAATTGATATTGTTCTGTATATTCAGGGGAAGATAAAAGGAAGGATCTTTCACT-3′

**Supplementary Text 2 *DoGMP2* putative promoter sequence:**

5′-TTTTAATATGCGGATGATGATGTCATGGATGATGTTCGAAATGACAATGAAATAATTGGCAATGACGATATTGCTGTTTTTGGAGGGGAATGTTTTGATGAACAAGGATTTATAGATATAACTTTTGATGCTTCAAATCTAGGAGACGACTTCTATATTTATTTTCATATTTATGTTTGAACTTTGAAGTTTGACTTGATATGAAATTATGAACATTTTGTTTGATGACAAAAGCTATGCATTTGTTTATATTTTATTGACTTATTTGATGCGACACTTATATTATGTGTTATTTGTTAATGTTTCATAAACTTAATTGATGTCATGTTAATGTTTCATAAACTTAATTGATGTCATGTTTATGTTATTTGAATTAGGAGTTATGAAAATAGAATGTATAATGAAGATATTACAGGAAATTTAATATTTATTTTATTTTTTTTAATAATATTATTATTTATTACATCATCCGATTCGATCTTGGTTCGAACAGACGAACCGGCCGAACCGTGAACCTCTCTTTCTACCAGTTCATTGTCCGGTCCAATTTTCAAACCCTTGAATAAATTAGAATAATTCTGATCGTCCCATCTCCGCACTAATAATTTATCATTTTATCCTTTTCTTCACGTTATACACACTCGTGAGTCCTGACGTCTTGATGTAGCTTCATTGGATTCGCCGATTGCATTCGAATTCCGTAGGCCCGGTTCTACCCTGGGCGGAAATCCGGGCCTACGGGCCGGTCCCACATCTCGGCCCGGTCCACGTTGTATTCATAGCCTTTCATCCGCGTTCTTACTCCTTAAAAGGCCAACGGAGCTGAAGCAGACCGGTATCGGTCCCTCCCGCCTCGTCTTCCGCGCACCCGTTGGCTTCTTCCTCCTCCTCCTAACTTACGTTACTTCCTCCAGAGCTGGAAAGCTAGGGTTAGCTTTGTTCCTCATGCGGTGAGCATCTTATTTTCTCGAGCTAGGGTTTATCGTTTCGGATCTGAGCA-3′

**Supplementary Text 3 *DoGMP3* putative promoter sequence:**

5′-AACATCTTCAAATTCCCTCAAACGTGAAGATTTCATTCACGAATAAAAGGAATGAAAATAGAAGTATTATGTTTTGAAAGATTTTTAATATTTGAAGCAGCCAAATGAGCCCTTAGATTGATGTTGAAGTCTTCTCTCACTTGCCAGCTAGTTAGACTACATTTGAGGCAGCTTATACTTTGCTGTTTTTGGTTTTTCAAAATTGCTTTCCAACAACAATTTCTATGATGCTTTCCCAATAATAACATTTATAAGACAAATTTTTTTTTCCAAATGTAGTTCAGAAGATATAATCCAAATAGATATTTTTATCATTCTTTGTTCTTTGCCACCACAAAAACGATTAAATTGCTATTTCAAATGAAACCTTAGTTCTATCTCTTGTAGGTAGTGTTGATTTACTGATTTCTTGAGAATGAGAAGCCCTAAGATGCATAACTATTTTTATGGAAAGCCCCTCAAAAAGAAATGATTCAGTTTTCACACAAAAAGAGTGAAGATATAAAATTATCATTTTCTAAAAATTTACAAGCAAGCAAATGAGCTGCAAGAGAAATTTCATATGCAGCCTTAAAACTAATGATATTTTAATCCTGCTGATGGTTTCAGGTGGAAGTGCAGCCCAGTCACAGTTAAATTACATGAGTAATTTATCATTTTGTAACTCTTAGATTTTGAGATTCTAATGTTGCCTTGGAGCTTTTTTTCGAATTGTGCTTGATCTTGGCTTTCCGTCTTTTTGATGCCTTCATGCCTAGACAATTTGTTTTTAATTTTTCCTTTATGGAATATGTTGTTTTAAGCTGGTAACCTTTCTTGGAAATATCAAATATGCTAGCTTGTCTGGTTCCTTGGTTAGCAAGTTTATTCCCTTCTATAGTCATAAGATGTTTTAGAAGTGCTGAAGAACACATGAATATATACTGCCTCTTTTTTATTTCATTTTCTGAGTGGTTAAACTTTTGATCCCAGTGAAAGGCAAGAAGTGGTATCTCTGAAA-3′

**Supplementary Table 1 Primers used for cloning *DoGMP* genes.**

| Primer name | Primer sequence |
| --- | --- |
| DoGPM1-3′F1 | 5′-ACATTGGGCAGCCTAAAGAC-3′ |
| DoGMP2-3′F1 | 5′-CTTCCCACTACCACAAATGC-3′ |
| DoGMP2-3′F2 | 5′-GAAGCCTTACAGTCAGCAAC-3′ |
| DoGMP3-3′F1 | 5′-GCTGCTCTGTAATGAAGGGAGT-3′ |
| DoGMP3-3′F2 | 5′-ACAAGGAGATCAAATCCAGCAT-3′ |
| DoGMP1-5′R1 | 5′-GATAAAGTGCGAACCTACGG-3′ |
| DoGMP1-5′R2 | 5′-TGCCCAATGTCCATCCAGAA-3′ |
| DoGMP2-5′R1 | 5′-TGGGCATCAAGCATTTGTGGT-3′ |
| DoGMP2-5′R2 | 5′-CATTTGTGGTAGTGGGAAGC-3′ |
| DoGMP3-5′R1 | 5′-AGAAAGGTTCACCAGAACCA-3′ |
| DoGMP3-5′R2 | 5′-ATATGAAGCTTATCTTCAAACT-3′ |

Gene-specific primers for gene cloning were designed by Primer 5.0.

**Supplementary Table 2 Primers used for cloning promoters.**

| Primer name | Primer sequence |
| --- | --- |
| GMP1-GSP1 | 5′-GCAAAATCCACTAGTGGTTTTGG-3′ |
| GMP1-GSP2 | 5′-GTGAAAGATCCTTCCTTTTATCT-3′ |
| GMP1-GSP3 | 5′-TAGAATTGCTTTGGCTAGAGCC-3′ |
| GMP2-GSP1 | 5′-TGCTGCAAGCAGAGATAGGATGA-3′ |
| GMP2-GSP2 | 5′-CGCAAGAGGAAACAACGGCTTCG-3′ |
| GMP2-GSP3 | 5′-ATCACAGCCACAACTCTCTCTT-3′ |
| GMP3-GSP1 | 5′-GCAGGCGGGTTCCAAACCCTCCA-3′ |
| GMP3-GSP2 | 5′-ATACCACTTCTTGCCTTTCACTG-3′ |
| GMP3-GSP3 | 5′-GAACCAGACAAGCTAGCATATT-3′ |

GSP primers were designed by Primer 5.0.

**Supplementary Table 3 Primers used for vector construction.**

| Primer name | Primer sequence |
| --- | --- |
| DoGMP1YFPF | 5′-CGAACGATAGCCATGGCTATGAAGGCACTAATTCTTGTCGG-3′ |
| DoGMP1YFPR | 5′-TGAGTCCGGACCATGGTCATAACAATCTCAGGTTTCAGAATGC-3′ |
| DoGMP1OF | 5′-GGACTCTTGACCATGGCTATGAAGGCACTAATTCTTGTCGG-3′ |
| DoGMP1OR | 5′-GTCAGATCTACCATGGTCATAACAATCTCAGGTTTCAGAATGC-3′ |

Primer pairs for vector construction were designed by Primer 5.0.

**Supplementary Table 4 Primers used for qRT-PCR.**

| Primer name | Primer sequence |
| --- | --- |
| AtCSLA1F | 5′-GAGATAACAGGAACGGCTACAA-3′ |
| AtCSLA1R | 5′-GACAGCGCGGATGAGATAAT-3′ |
| AtCSLA10F | 5′-GTCTTGCCTTCCTCTCTCTAATG-3′ |
| AtCSLA10R | 5′-AGCTTATCCTCGCACCATTTAT-3′ |
| AtCSLA11F | 5′-GATGACTAGGATGCAGGAGATG-3′ |
| AtCSLA11R | 5′-GCAGTCCCATTAAAGCCAAAG-3′ |
| AtCSLA14F | 5′-CCAGCTACCATCACTATCCTAAAC-3′ |
| AtCSLA14R | 5′-ACTCCTATGCATCGCCATTAC-3′ |
| AtCSLA15F | 5′-AGAGGCGAGAGAACAGAAATG-3′ |
| AtCSLA15R | 5′-GGCTGGAAATCAGCATCAAAG-3′ |
| AtCSLA2F | 5′-CCGCCGGAATATGGAGAATAG-3′ |
| AtCSLA2R | 5′-AGGTCACCGAGGTAGAGAAA-3′ |
| AtCSLA3F | 5′-CCTCGATCACTCCATCTTCTTG-3′ |
| AtCSLA3R | 5′-GCCTCTAGTAACCCGATGAATG-3′ |
| AtCSLA7F | 5′-GGTTGGAGCGTACCTGTTATT-3′ |
| AtCSLA7R | 5′-AGAAGGCCACTGACTGAAAC-3′ |
| AtCSLA9F | 5′-GCTGGTGGTTGGAAAGATAGA-3′ |
| AtCSLA9R | 5′-CCTTCAAAGAACCGAGGTACAA-3′ |
| DoGMP1RTF | 5′-CTCAGTCCTCGACCTTATCGAAT-3′ |
| DoGMP1RTR | 5′-ACTGTAGAATGCCATCCAATGAT-3′ |
| AtUBQ10F | 5′-GATCTTTGCCGGAAAACAATTGGAGGATGGT-3′ |
| AtUBQ10R | 5′-CGACTTGTCATTAGAAAGAAAGAGATAACAGG-3′ |

Primer pairs for qRT-PCR were designed by online Primerquest software (<http://www.idtdna.com/Primerquest/Home/Index>). The gene-specific primers for *DoGMP1* were designed according to the sequence alignment of *DoGMP1* and *GMP* genes from *Arabidopsis thaliana*.
